# Editorial: Reducing cardiovascular disease mortality and morbidity: implementing cost-effective and sustainable preventive interventions

**DOI:** 10.3389/fcvm.2023.1236210

**Published:** 2023-06-20

**Authors:** Shanthi Mendis, Ian Graham, Jagat Narula

**Affiliations:** ^1^ Department of Global Health, Senior Adviser (Formerly WHO), The Geneva Learning Foundation, Geneva, Switzerland; ^2^ Department of Cardiology, Trinity College Dublin, Dublin, Ireland; ^3^ Department of Health Science, University of Texas Health Science Center at Houston, Houston, TX, United States

**Keywords:** cardiovascular disease, costeffective, sustainable, universal coverage, primary healthcare

**Editorial on the Research Topic** Reducing cardiovascular disease mortality and morbidity: implementing cost-effective and sustainable preventive interventions

The global burden of cardiovascular diseases (CVD) is growing due to inadequate prevention and control of behavioral and metabolic risk factors, population growth, and aging (1). The CVD burden is highest in low-and middle-income countries with fragile health systems and financial and human resource constraints. There are very effective interventions that are affordable to all countries for addressing the cardiovascular epidemic (2). However, these interventions still need to be widely utilized (3). They include (1) public health policies to reduce behavioral risk factors, (2) access to integrated management of cardiovascular risk factors (hypertension, diabetes, hyperlipidemia, and other behavioral risk factors) and the mitigation of cardiovascular risk through a primary health care approach, and (3) universal health coverage of very cost-effective cardiovascular interventions including secondary prevention of heart attacks and strokes. The main aim of this Research Topic is to collate original research articles and reviews that provide insight into structural and contextual factors that impede the prioritization and uptake of affordable and sustainable interventions for CVD prevention and control.

Moyá-Amengual et al. evaluate the prevalence of elevated pulse pressure in an adult population in primary care and its association with other vascular risk factors, subclinical target organ damage, and CVD. Elevated pulse pressure was present in a quarter of the sample, and the prevalence increased with age. Raised pulse pressure was more frequent in men, patients with hypertension, CVD, and other target organ damage. These findings add to the body of evidence demonstrating that pulse pressure is an independent cardiovascular risk factor in the population over 60 years.

A healthy lifestyle, including physical activity, is featured as a key intervention for CVD prevention in this special issue. Su et al. investigate the associations of a healthy lifestyle with the subsequent development of CVD. During a 10-year follow-up of 51,929 participants, adherence to a healthy lifestyle pattern was associated with a lower risk of CVD, but this benefit was not as pronounced among normotensives as among hypertensives.

Zhou et al. conduct a meta-analysis to evaluate the effect of exercise on vascular function in patients with hypertension. Aerobic, resistance, and high-intensity intermittent exercise all significantly improved systolic blood pressure, diastolic blood pressure, and endothelium-dependent flow-mediated dilatation but not pulse wave velocity. More research is needed to investigate which exercise modality can improve hypertension-mediated vascular dysfunction and vascular sclerosis, and provide more effective exercise programs for hypertensive patients.

Eser et al. report an observational, longitudinal study to evaluate adherence to physical activity recommendations after percutaneous coronary interventions for coronary syndromes. They find that the moderate to vigorous physical activity recommendations of the World Health Organization physical activity guidelines can be fulfilled easily through activities of daily living without any planned or structured exercise. Future studies are needed to clarify how the recommendations are actionable for patient benefit.

This special issue includes the clinical trial protocol of a prospective study by Gonzalez-Jaramillo et al. that investigates the associations of objectively measured physical activity with major adverse cardiac events and mortality at 1-year follow-up after percutaneous coronary intervention.

Rosende et al. describe the results of a multi-country study designed for stepwise quality improvement of cardiovascular risk management, including hypertension control, using a primary health care approach. The aim was to identify improvement areas, reveal the challenges, and extend best practices for hypertension control and CVD risk management in primary health care. The study highlights many challenges to the prevention and control of CVD, for example, in the organization of health services including barriers to drug titration by non-physician health workers such as nurses and pharmacists. The authors also report a recurring concern from implementing countries that the treatment protocol of the study seemed too focused primarily on hypertension, although hypertension and diabetes have overlapping risk factors that lead to common pathways of complications and target organ damage. These findings highlight the dire need to promote a more integrated approach to address cardiovascular risk factors (hypertension, diabetes, hyperlipidemia, tobacco use, physical inactivity, and overweight) (4), secondary CVD prevention, and other common comorbidities such as depression and chronic kidney disease, particularly in primary care.

Indeed, the importance of using an integrated approach to address CVD that encompasses comorbidities is underscored by the study of Shen et al. They assess the relationship between the level of a person’s depression and their risk of coronary heart disease, stroke, and all-cause and cardiovascular mortality, utilizing data from the United States National Health and Nutrition Examination Survey. They report a statistically significant association between depression and increased risk of coronary heart disease and stroke.

In addition to using integrated approaches in primary care, minimizing barriers to health system organization and access to essential medicines is critical for improving health outcomes. Pang et al. conduct a study in Yunnan Province, China, to assess the feasibility and impact of a nationwide healthcare service. They evaluate the accessibility and efficacy of basic public health care services by analyzing variables such as blood pressure, body mass index, lifestyle modification, and cardiovascular risk factors. The impact of the national basic public health care services program was evident in lowering risk factors for cardiovascular diseases, promoting healthy lifestyles, lowering blood pressure, increasing medication adherence, and the better control rate of hypertension.

Guo et al. analyze the effect of a full coverage policy of essential medicines on medication adherence. For patients with hypertension and diabetes, the full coverage policy of essential medicines in Taizhou, China, resulted in an increase in adherence to antidiabetic and antihypertensive medicines. These findings suggest that policymakers should consider removing cost-sharing for essential medicines as a promising strategy to improve medication adherence in people with non-communicable diseases, particularly in socially disadvantaged groups.

This Research Topic illustrates the breadth of research being undertaken regarding lifestyle, metabolic risk factors, primary health care, health systems, access to medicines, and Universal Health Coverage, in the prevention and management of CVD.
